# Supplementary material for: HOX gene expression in the developing human spine
Source: Nat Commun. 2024 Nov 20;15:10023. doi: 10.1038/s41467-024-54187-0 (PMC11579336; doi:10.1038/s41467-024-54187-0)
Supplement: Supplementary file 1 — Supplementary Information [file 41467_2024_54187_MOESM1_ESM.pdf]

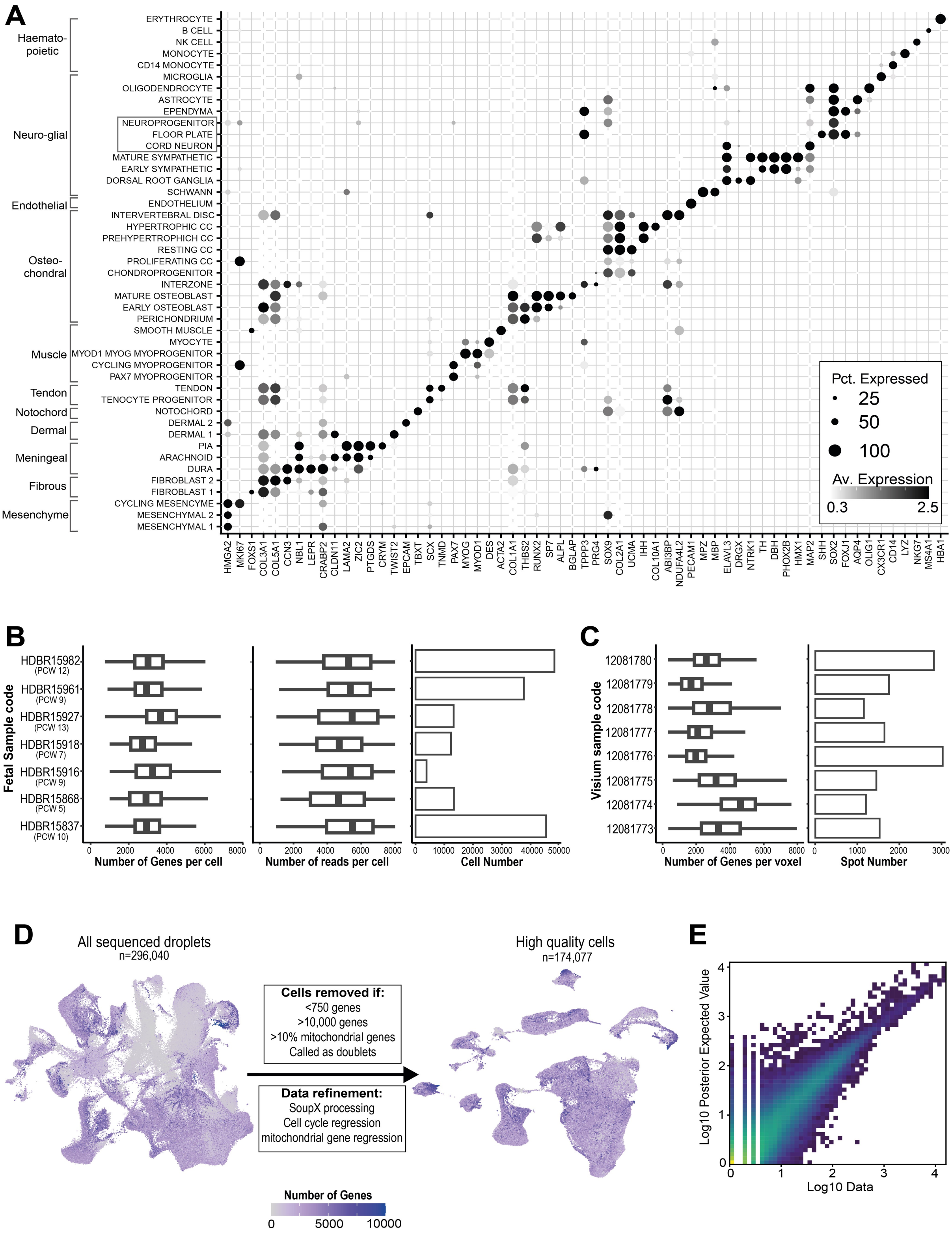

Supplementary Figure 1. Quality control and spinal atlas marker genes

(A) Dotplot of mean log-normalised expression values of marker genes amongst cells of the fetal spine. Point size shows the percentage of cells with non-zero expression. Grey box denotes cells sub-clustered for analysis in Fig.2. For references for marker genes see Supplementary Data 1. CC, chondrocyte; Pct. Exp, percentage of expressing cells; Av. Exp, average expression in cell type.

(B) Quality control plots for single cell data. Left panel - Number of genes expressed per cell in each fetal sample. Middle panel - number of sequencing reads per cell for each sample. Right plot- number of cells captured from each fetal sample

(C) Quality control plots for Visium spatial transcriptomics data. Left panel - Number of genes expressed in each capture area. Right plot- number of voxels sequenced from each capture area.

(D) Uniform manifold approximation and projection (UMAP) plot visualising single cell RNA sequencing data with (right panel) and without (left panel) quality control measures.

(E) Scatter plot showing reconstruction accuracy of cell2location data

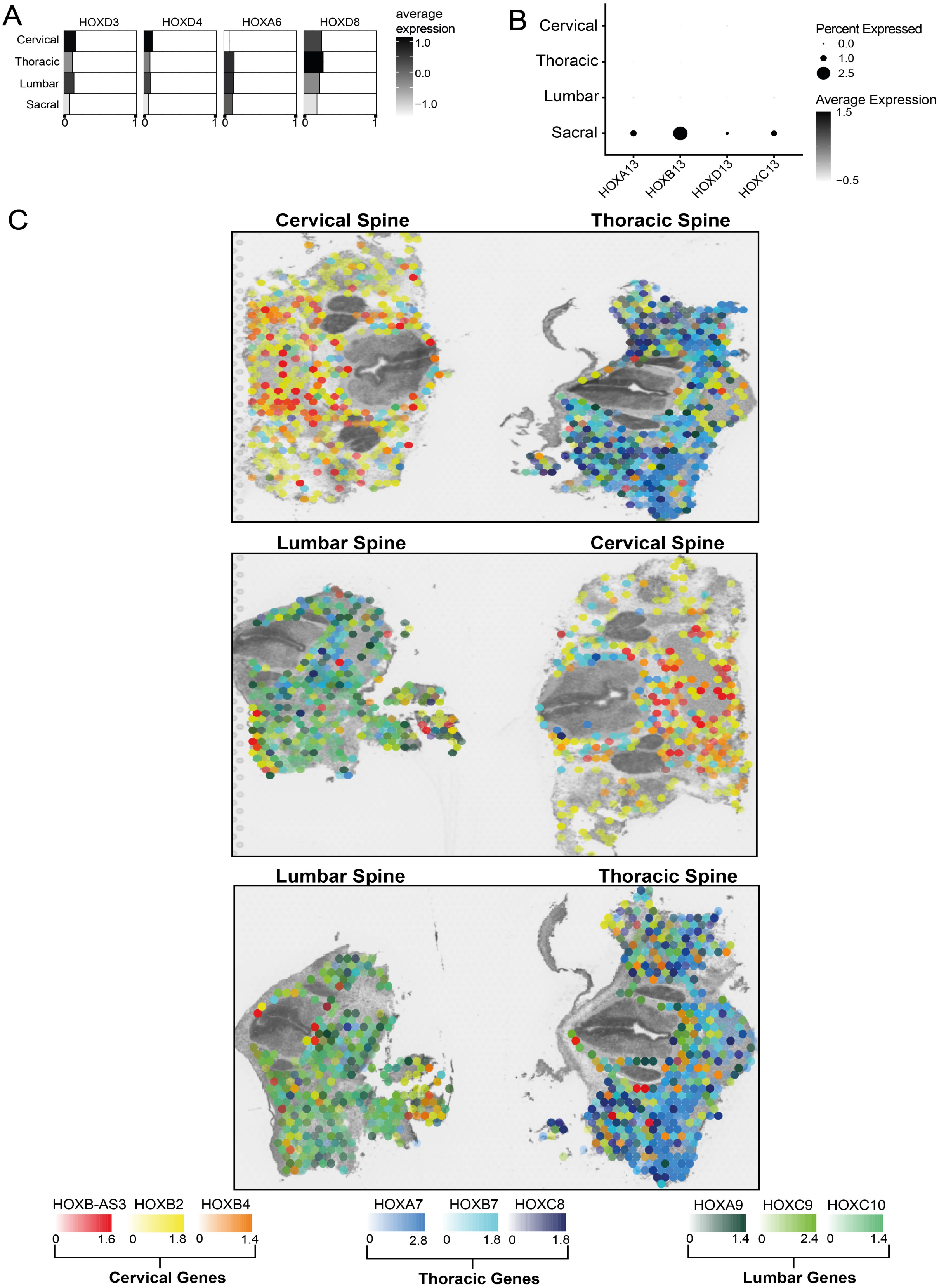

Supplementary Figure 2. HOX gene filtering and spatial transcriptomic data

(A) Barplots quantifying log-normalised expression across spinal segments of HOX genes with idiosyncratic expression patterns in tenocytes. Bar length corresponds to the fraction of cells with non-zero expression.

(B) Dotplot of mean log-normalised expression values of group 13 HOX genes amongst stationary cell type cells. Point size shows the percentage of cells with non-zero expression.

(C) Spatial heatmaps of all PCW7 spatial transcriptomic samples showing log-normalised values of HOX gene expression

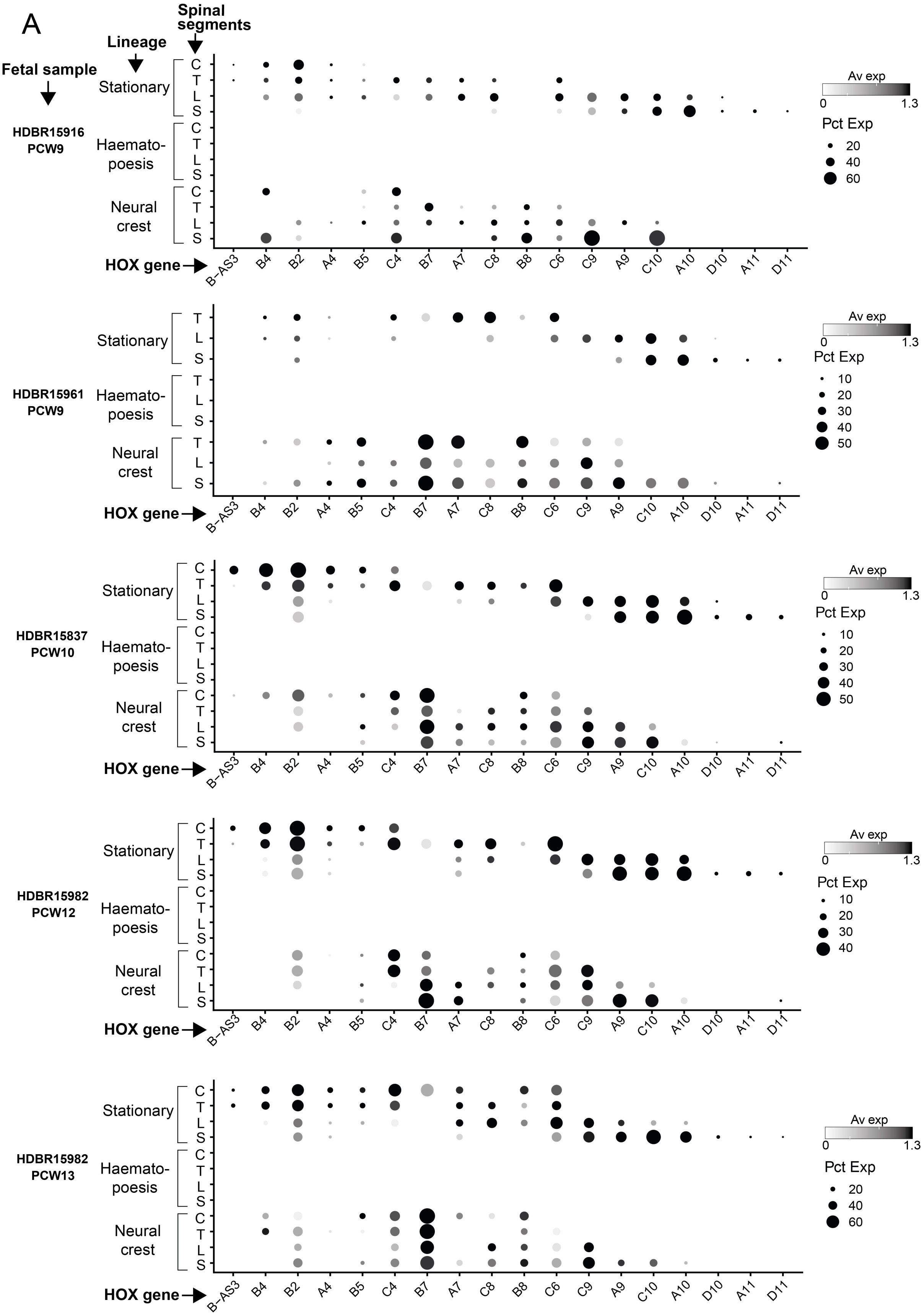

Supplementary Figure 3. HOX genes in different cell types across fetal samples

(A) Dotplot of mean log-normalised expression values of HOX genes amongst stationary, haematopoietic and neural cells across different fetal samples. Point size shows the percentage of cells with non-zero expression. PCW, post-conception weeks; C, cervical; T, thoracic; L, lumbar; S, sacral; Pct Exp, Percentage of cells expressing; Av exp, Average expression in group.

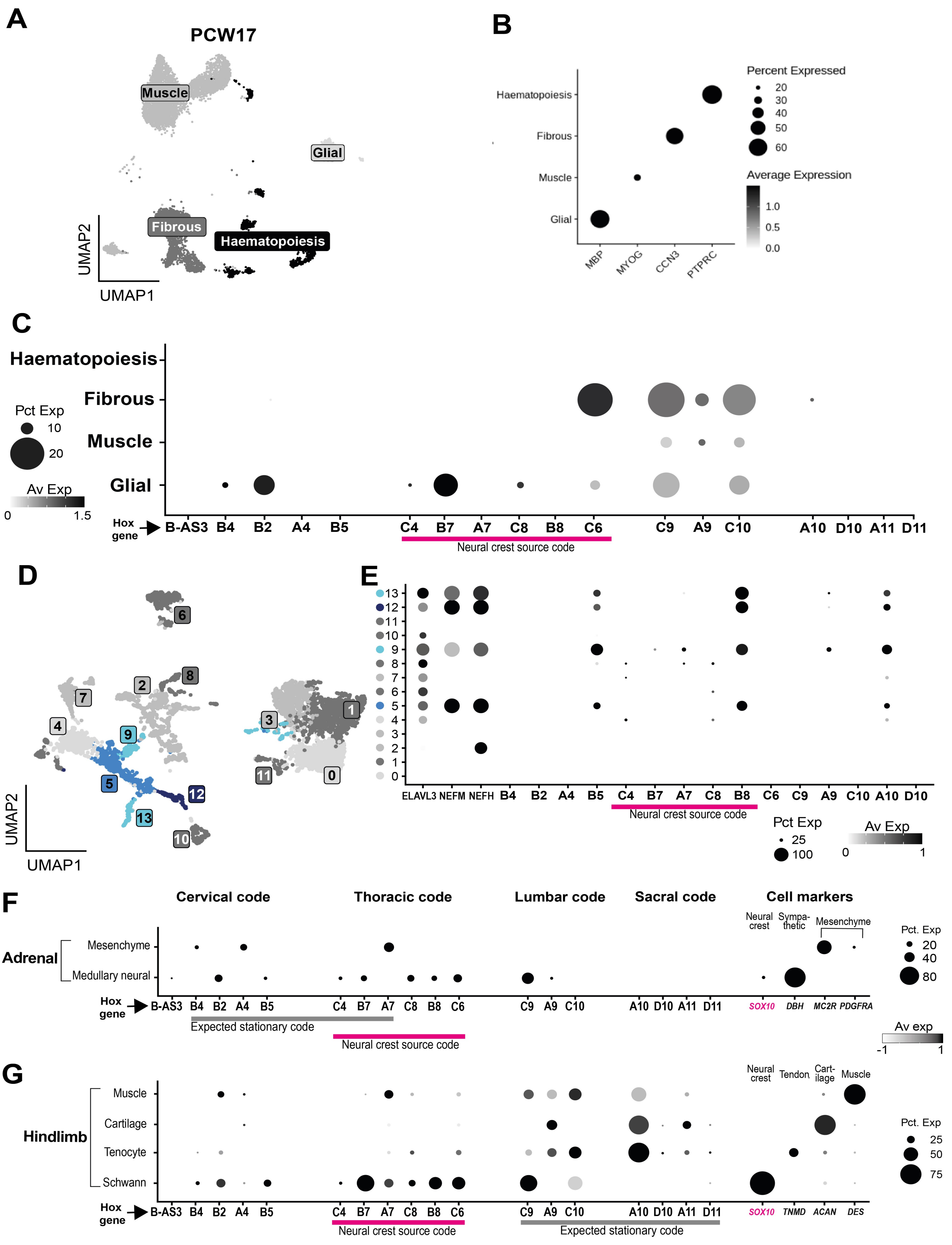
A
PCW17
Muscle
Glial
Fibrous
Haematopoiesis
UMAP2
UMAP1
B
Percent Expressed
20
30
40
50
60
Average Expression
1.0
0.5
0.0
Haematopoiesis
Fibrous
Muscle
Glial
MBP
MYOG
CCN3
PTPRC
C
Haematopoiesis
Fibrous
Muscle
Glial
Pct Exp
10
20
Av Exp
0
1.5
Hox gene
B-AS3 B4 B2 A4 B5 C4 B7 A7 C8 B8 C6 C9 A9 C10 A10 D10 A11 D11
Neural crest source code
D
6
2
8
7
4
9
3
1
5
12
13
11
0
10
UMAP2
UMAP1
E
13
12
11
10
9
8
7
6
5
4
3
2
1
0
ELAVL3 NEFM NEFH B4 B2 A4 B5 C4 B7 A7 C8 B8 C6 C9 A9 C10 A10 D10
Neural crest source code
Pct Exp
25
100
Av Exp
0
1
F
Cervical code
Thoracic code
Lumbar code
Sacral code
Cell markers
Neural crest
Sympa-thetic
Mesenchyme
Adrenal
Mesenchyme
Medullary neural
Hox gene
B-AS3 B4 B2 A4 B5 C4 B7 A7 C8 B8 C6 C9 A9 C10 A10 D10 A11 D11
SOX10 DBH MC2R PDGFRA
Expected stationary code
Neural crest source code
Pct. Exp
20
40
80
Av exp
-1
1
G
Neural crest
Tendon
Cart-ilage
Muscle
Hindlimb
Muscle
Cartilage
Tenocyte
Schwann
Hox gene
B-AS3 B4 B2 A4 B5 C4 B7 A7 C8 B8 C6 C9 A9 C10 A10 D10 A11 D11
SOX10 TNMD ACAN DES
Neural crest source code
Expected stationary code
Pct. Exp
25
50
75

Supplementary Figure 4. HOX gene expression in 2nd trimester and adult lumbar dorsal root ganglia, the developing adrenal gland and developing hindlimb.

(A) Uniform manifold approximation and projection of 9,555 PCW17 lumbar fetal spinal cells, shaded by category.

(B) Dotplot of mean log-normalised expression values of classical marker genes amongst cells depicted in (A). Point size shows the percentage of cells with non-zero expression

(C) Dotplot of mean log-normalised expression values of HOX genes amongst cell clusters depicted in A. Point size shows the percentage of cells with non-zero expression. Pct Exp, Percentage of cells expressing; Av exp, Average expression in group.

(D) Uniform manifold approximation and projection of 6,307 adult L4&5 dorsal root ganglia cells, shaded by category. Blue shades represent HOX-expressing cells.

(E) Dotplot of mean log-normalised expression values of neuronal marker genes and HOX genes amongst cell clusters depicted in D. Point size shows the percentage of cells with non-zero expression. Y axis dot colour corresponds to colour on UMAP in D. Pct Exp, Percentage of cells expressing; Av exp, Average expression in group.

(F) Dotplot of mean log-normalised expression values of neuronal and mesenchymal marker genes and HOX genes amongst cells of the fetal adrenal gland. Point size shows the percentage of cells with non-zero expression. Pct Exp, Percentage of cells expressing; Av exp, Average expression in group.

(G) Dotplot of mean log-normalised expression values of neuronal, tenocyte, cartilage and muscle marker genes and HOX genes amongst cells of the fetal hindlimb. Point size shows the percentage of cells with non-zero expression. Pct Exp, Percentage of cells expressing; Av exp, Average expression in group.

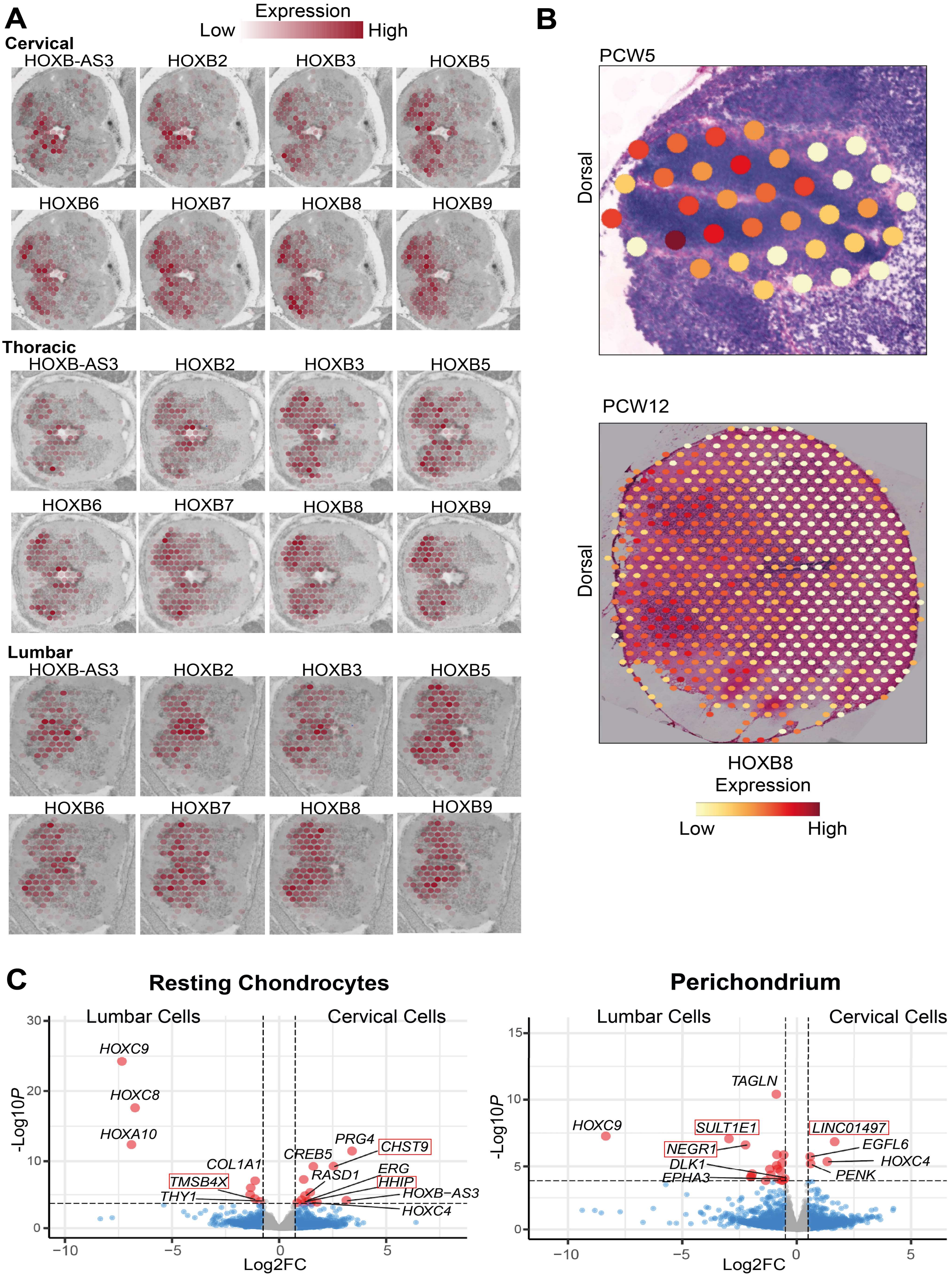

Supplementary Figure 5. HOXB gene expression in the fetal spinal cord.

(A) Spatial heatmaps of all HOXB group genes expressed in the Post-conception week 9 fetal cord across three anatomical levels.

(B) Spatial heatmaps of all HOXB8 expression in the developing cord at Post-conception week 5 and 12. PCW, post-conception week.

(C) Volcano plots of differentially expressed genes between lumbar and cervical regions in perichondrium and resting chondrocytes. Red boxes indicate region-specific genes discussed in the text. Red dots represent genes that are significantly differentially expressed. Blue and grey dots represent genes not reaching significance.
