# Supplementary material for: HOX gene expression in the developing human spine
Source: Nat Commun. 2024 Nov 20;15:10023. doi: 10.1038/s41467-024-54187-0 (PMC11579336; doi:10.1038/s41467-024-54187-0)
Supplement: Supplementary file 3 — Description of Additional Supplementary Files [file 41467_2024_54187_MOESM3_ESM.pdf]

Description of Supplementary Information

Supplementary Data 1

Quality control of single cell and spatial transcriptomic data

Supplementary Data 2

References for marker genes used in single cell annotation (Extended Data Figure 1 and Extended Data Figure 2) and segment specific genes (Extended Data Figure 7)

Supplementary Data 3

The rostrocaudal HOX code in static and neural crest-derived cell types. Wilcoxon rank-sum test (two-sided) corrected for multiple comparisons.

Supplementary Data 4

HOX expression by static cell types, across regions and developmental time. Wilcoxon rank-sum test (two-sided) corrected for multiple comparisons.

Supplementary Data 5

HOX gene expression by different cell types in the fetal limb, gut and adrenal gland. Wilcoxon rank-sum test (two-sided) corrected for multiple comparisons.

Supplementary Data 6

References for genes that varied with anatomical location in perichondrium and resting chondrocytes (Fig S7C). Wilcoxon rank-sum test (two-sided) corrected for multiple comparisons.

Supplementary Data 7

List of in-situ sequencing and RNA-in situ hybridisation probes
